# Biases in COVID-19 vaccine effectiveness studies using cohort design

**DOI:** 10.3389/fmed.2024.1474045

**Published:** 2024-10-30

**Authors:** Suneth Agampodi, Birkneh Tilahun Tadesse, Sushant Sahastrabuddhe, Jean-Louis Excler, Jerome Han Kim

**Affiliations:** ^1^Innovation, Initiatives and Enterprise Development Unit, International Vaccine Institute, Seoul, Republic of Korea; ^2^Section of Infectious Diseases, Department of Internal Medicine, Yale University School of Medicine, New Haven, CT, United States; ^3^Epidemiology, Public Health, and Impact Unit, International Vaccine Institute, Seoul, Republic of Korea; ^4^International Vaccine Institute, Seoul, Republic of Korea; ^5^College of Natural Sciences, Seoul National University, Seoul, Republic of Korea

**Keywords:** COVID-19, vaccine effectiveness, cohort studies, biases, misclassification bias, healthy user bias, healthy vaccinee effect, differential depletion of susceptibility bias

## Abstract

Observational studies on COVID-19 vaccine effectiveness (VE) have provided critical real-world data, informing public health policy globally. These studies, primarily using pre-existing data sources, have been indispensable in assessing VE across diverse populations and developing sustainable vaccination strategies. Cohort design is frequently employed in VE research. The rapid implementation of vaccination campaigns during the COVID-19 pandemic introduced differential vaccination influenced by sociodemographic disparities, public policies, perceived risks, health-promoting behaviors, and health status, potentially resulting in biases such as healthy user bias, healthy vaccinee effect, frailty bias, differential depletion of susceptibility bias, and confounding by indication. The overwhelming burden on healthcare systems has escalated the risk of data inaccuracies, leading to outcome misclassifications. Additionally, the extensive array of diagnostic tests used during the pandemic has also contributed to misclassification biases. The urgency to publish quickly may have further influenced these biases or led to their oversight, affecting the validity of the findings. These biases in studies vary considerably depending on the setting, data sources, and analytical methods and are likely more pronounced in low- and middle-income country (LMIC) settings due to inadequate data infrastructure. Addressing and mitigating these biases is essential for accurate VE estimates, guiding public health strategies, and sustaining public trust in vaccination programs. Transparent communication about these biases and rigorous improvement in the design of future observational studies are essential.

## 1 Introduction

The COVID-19 pandemic has triggered numerous vaccine studies, with 1,263 vaccine trials (all phases) registered in the World Health Organization's International Clinical Trials Registry Platform (WHO ICTRP) for more than 250 vaccine candidates (1). These vaccine trials involved more than 80 countries within the first 2 years of the pandemic (99). Due to the urgency of the pandemic, authorities were searching for rapid and reliable data on vaccine efficacy and robust real-world data on vaccine effectiveness (VE) to develop evidence-based vaccination strategies (2).

While randomized, placebo-controlled clinical trials provide robust efficacy and safety data, their value is limited by stringent inclusion criteria, lack of representativeness of diverse populations, and controlled environments that do not reflect local epidemiology or programmatic challenges. Thus, observational studies became crucial due to their rapid implementation, cost-effectiveness, and flexibility in integrating pre-existing data sources (3). These studies can capture the performance of vaccines across diverse populations, address operational issues, and inform sustainable, contextually appropriate vaccination policies using real-world evidence (4).

The landscape of observational studies on the effectiveness of COVID-19 vaccination shows that more than 2200 observational studies were published directly or indirectly referring to VE by the end of 2022 (5). Despite their importance, observational studies are prone to different biases, some being exemplified by COVID-19 pandemic-related factors (6). Recognizing the importance of these errors in VE studies, the WHO prepared interim guidelines for evaluating COVID-19 VE in 2021 (7) and a revision in 2022 (8). These documents have comprehensively outlined studies, errors, and measures to overcome those possible challenges. Despite these guidelines, the biases in observational studies on VE prevailed. An evaluation of those biases and a closer look into how these predicted biases in VE were observed in COVID-19 offers a unique opportunity to design better vaccine effectiveness studies in future pandemics (9). Test-Negative Design (TND), a case-control approach for estimating VE, involves comparing the odds of vaccination among individuals who test positive or negative for the disease. It was widely used during the early pandemic and extensively discussed (10–16). Hence, this paper explores common biases in cohort studies (Table 1), the most common type of studies used for COVID-19 VE assessments (17), to guide future researchers in planning VE studies.

**Table 1:** Biases in cohort studies investigating COVID-19 vaccine effectiveness (VE).

**Bias**	**Description**
Socioeconomic and demographic bias	Unequal access to vaccines due to socioeconomic and demographic disparities can inflate VE estimates, as vaccinated individuals may have better overall health outcomes.
Healthy user bias	This bias occurs when individuals who get vaccinated are also more likely to engage in other health-promoting behaviors, leading to an overestimation of VE.
Healthy vaccinee effect	The tendency for vaccinated individuals to be generally healthier can result in an overestimation of VE, as they are less likely to experience severe disease outcomes.
Frailty bias	Frailty bias occurs when individuals with poorer health or more comorbidities are more likely to get vaccinated, leading to an underestimation of VE.
Confounding by indication	Confounding by indication arises when patients with a higher perceived or known risk of severe disease are more likely to get vaccinated, affecting VE estimates due to differences in baseline health risks.
Differential depletion of susceptibles bias	This bias happens when highly susceptible individuals are disproportionately removed from the population over time, making it seem as if VE is declining.
Attrition bias	Attrition bias occurs when there are systematic differences between individuals who included in the final analysis and those who loss to follow-up, leading to non-representative samples and potentially biased VE estimates.
Immortal time bias	Immortal time bias arises when an “immortal” period during which the outcome cannot occur is incorrectly included in the vaccinated group, falsely enhancing VE.
Misclassification bias	Misclassification bias occurs when individuals or events are incorrectly categorized regarding vaccination status or disease outcome, leading to inaccurate VE estimates.
Non-differential misclassification of outcomes	This bias happens when the misclassification rate is similar across groups, generally biasing results toward the null and diluting the observed effect size.
Differential misclassification of outcomes	Differential misclassification occurs when the likelihood of misclassification differs between groups, leading to either an overestimation or underestimation of VE.
Case counting window bias	Case counting window bias occurs when the time frame for counting cases is misaligned between vaccinated and unvaccinated groups, leading to biased VE estimates.
Waning immunity bias	Waning immunity bias arises when the natural decrease in immune response over time is not accounted for, creating the perception of declining VE in long-term studies.

## 2 Biases due to differential vaccination in populations

**Figure 1:**
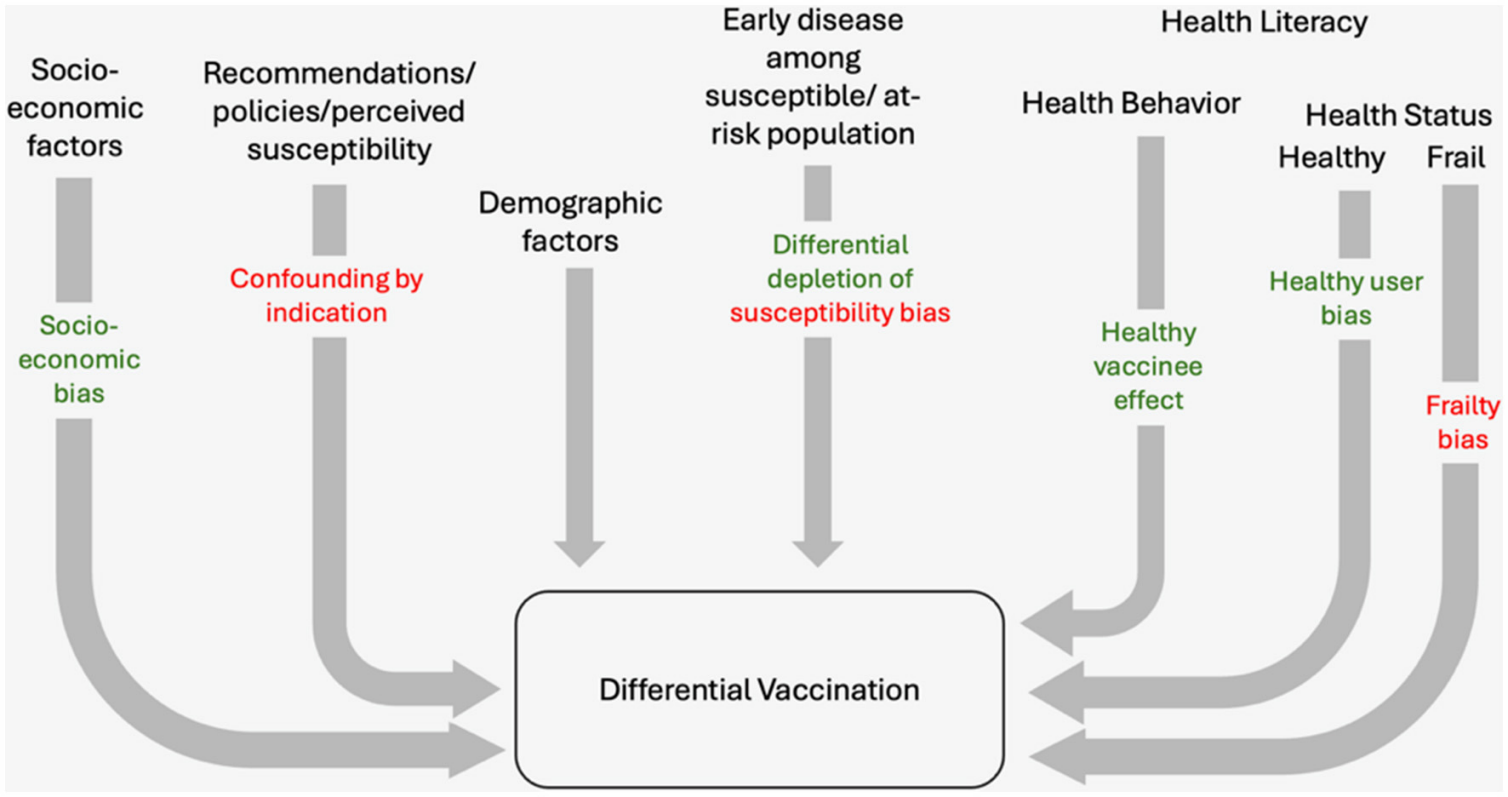
Differential COVID-19 vaccination leading to inaccurate estimates of vaccine effectiveness in cohort studies. The colored labels show the specific names given to the biases that resulted in specific differential vaccinations. Green, overestimation of VE; Red, underestimation of VE.

In COVID-19 VE studies employing a cohort design, individuals vaccinated through mass immunization programs are enrolled as cohort participants. If the vaccinated population systematically differs from the unvaccinated population, the estimated VE does not reflect the true VE. Biased estimates of VE occur when the participants selected for the study do not represent the general population, leading to systematic differences in the association observed between exposure and outcome among those selected and those eligible. These disparities lead to a bias often called “selection bias.” However, the type of biases in VE studies using cohort design that occurs due to differential vaccinations are not due to conditioning on common effect but resulting from the existence of common causes of exposure and outcome, which could be classified as confounding (18). Despite the classification used (as confounding or bias), a thorough assessment of how these systematic differences occur is crucial to understanding biases in COVID-19 VE studies (Figure 1).

### 2.1 Coverage-dependent bias in COVID-19 VE estimates

During the initial phase of vaccination campaigns, when coverage was low, the vaccinated group systematically differed from the unvaccinated group. These differences can cause the VE estimate to be underestimated or overestimated, depending on underlying reasons such as country-specific priorities and selective vaccination practices. This bias is sometimes referred to as “early vaccine bias” (8). Similarly, when vaccine coverage is high, unvaccinated individuals systematically differ from the vaccinated or general populations. Personal beliefs, health promotion practices, contraindications, or access issues can all contribute to inaccurate VE estimates among unvaccinated people in high-coverage settings. The Weekly COVID-19 Vaccination Dashboard of the Center for Disease Control (CDC) clearly showed these differences since 2021, when coverage was low and high (19). Any VE study conducted during these phases may be biased. This bias likely influenced many early COVID-19 VE studies when vaccination coverage was low, and it continues to affect studies as coverage increases beyond a certain level. Many biases described under “selection” biases could be part of this broader bias.

### 2.2 Biased selection due to socioeconomic status and demographic factors

Despite the massive production of COVID-19 vaccines, data worldwide show socioeconomic and demographic disparities leading to unequal availability, access, and affordability of vaccination (20). These factors separate those vaccinated from those who are not. The observation of this disparity is universal (21–24) and operates at global as well as national and subnational levels (25). Early in COVID-19 immunization programs, US data reported that Black and Hispanic ethnic groups and those who live in rural areas were less likely to be vaccinated (26), and these categories also have lower access and affordability to general healthcare, resulting in worse health outcomes. Data from more than 35 million people in the UK shows that the lower socioeconomic groups were less likely to get vaccinated (19). These socioeconomic disparities and inequalities systematically differentiate the vaccinated group, which typically has a lower infection risk and better disease outcomes. This usually leads to inflation of VE estimates, which may not apply to the general population.

A similar bias may operate in the opposite direction. Certain demographics, especially the young population, reported higher levels of vaccine hesitancy and declining to get vaccinated in some settings (27, 28). Our World in Data shows that in many countries, the lowest proportion of vaccination is among those between 18 and 25 years of age (29). Even though not vaccinated, this group is generally young, healthy, and less susceptible to severe complications and deaths. Combined data from Europe and the US shows that among the adult population, the lowest case fatality rate is among this age group (30). If this occurs in a specific community where VE studies are conducted, the estimates could be lower than expected due to demographic differences in the vaccinated and unvaccinated groups. In contrast, having elderly and more vulnerable populations in vaccinated cohorts might reduce the VE estimate. Thus, this bias could affect the VE estimate in both directions.

### 2.3 Healthy user bias

Healthy user bias is a broader concept where individuals who engage in one health-promoting behavior (such as vaccination) are more likely to engage in other health-promoting behaviors. This bias can lead to an overestimation of VE. If vaccinated individuals are also more likely to follow other preventive measures such as mask-wearing and social distancing, the observed benefits may be due to these combined behaviors rather than the vaccine alone. Healthy user bias typically affects prospective cohort designs on VE, where the prospective vaccine campaign participants primarily consist of those with healthy behaviors. One of the early COVID-19 VE studies from Hungary showed that the crude mortality rate was 5.3 per 100,000 person days among the unvaccinated group compared to 4.0 among the vaccinated group even during the non-endemic period, showing that the two groups may be systematically different and the vaccinated group is generally healthier (31). A retrospective cohort study using 6,974,817 individuals from December to June 2020 shows that recipients of three COVID-19 vaccines had lower non-COVID-19 mortality risk than their comparator groups. For mRNA vaccines, the adjusted hazard ratios of dose 1 and dose 2 ranged from 0.38 to 0.48 (32). Another early analysis from Milwaukee showed that unvaccinated persons had over twice the risk of non-COVID-19 natural death than the vaccinated (33). Many subsequent studies show reduced hospitalizations and deaths due to other causes in the vaccinated group, confirming the presence of healthy user bias (34, 101). In studies where all-cause mortality is considered as the outcome for VE calculation, this could have a more profound effect, where healthy behaviors may affect the risk of deaths due to other causes as well.

### 2.4 Healthy vaccinee effect

The “healthy vaccinee effect” is a related but distinct concept from “healthy user bias” in observational studies on VE. In these studies, individuals who choose to get vaccinated are generally healthier than those who do not (35). This can lead to overestimating VE since healthier individuals are naturally less likely to experience severe disease outcomes independent of the VE. Part of this effect could be observed in COVID-19 studies where the overall mortality and hospital admission rates are lower during the first few weeks after vaccination (36). The age-standardized all-cause mortality rate from the UK in 2021 shows that within the first 3 weeks following the first dose of vaccination, the death rate was 795.2 per 100,000 people. This rate increased to 1,232.2 per 100,000 people after the initial 3 weeks. A similar pattern was observed with the second dose, where the death rate was 471 per 100,000 people within the first 3 weeks, rising to 850 per 100,000 people after 3 weeks (37). The lower all-cause death rate within the first 3 weeks of vaccination is likely due to the fact that those vaccinated are not acutely ill at the time of vaccination, suggesting the indirect presence of the Healthy Vaccine Effect. Xu et al. reported that age, sex, and race/ethnicity groups adjusted non-COVID-19 related mortality rates among COVID-19 vaccinees were lower than those among comparators for the first three COVID-19 vaccines licensed in the USA. After the first dose, the adjusted hazard ratios (aHRs) were 0.46 for BNT162b2, 0.41 for the mRNA-1273 vaccine, and 0.55 for Ad26.COV2.S (32). Ostropolets and Hripcsak extensively analyzed this effect using a retrospective cohort design based on electronic health records. It showed that even after adjustment for many health-related variables, the vaccinated group had low overall health-seeking and hospital admission within the first few weeks of vaccination (38).

### 2.5 Frailty bias

The opposite of the healthy vaccine effect is known as the “frailty bias” or “unhealthy vaccine effect.” The impact of frailty on the outcome of COVID-19 is well-known, and low vaccine effectiveness among frail vaccine recipients is reported in many studies. For example, Meeraus et al. analyzed over 4.5 million AZD1222 vaccine recipients and noted that VE against hospitalization was >90% in the lowest multimorbidity quartile compared to 80% in the highest quartile. Further, among the elderly who are 'fit,' the VE was 86.2%, whereas it was 72% among the frail. VE against hospitalization was lowest in immunosuppressed individuals (65%) (39). However, frailty could be a selection bias as well. This bias occurs when individuals who are more likely to get vaccinated are those with poorer health or more comorbidities, leading to an underestimation of VE. In this scenario, the vaccinated group appears to have worse health outcomes not due to the vaccine's ineffectiveness but because they were already in poorer health than the unvaccinated group.

### 2.6 Confounding by indication

Confounding by indication occurs when the reason for vaccination is related to the patient's health status or risk of the outcome being studied. Patients with a higher perceived or known risk of severe COVID-19 might be more likely to get vaccinated, such as older adults or those with comorbidities. An analysis of vaccine policies from 185 countries shows that, except in the Western Pacific region, all other regions prioritized clinically vulnerable and elderly populations in their vaccine rollout (39). These subgroups have a higher baseline risk for unfavorable outcomes, which can confound observed VE because their health outcomes might differ from those not vaccinated regardless of the vaccine's true effectiveness. This can lead to overestimating or underestimating VE due to differences in baseline risk between treated and untreated groups. The bias was well-known to researchers yet common in published literature (40).

While both frailty bias and confounding by indication involve differential selection for vaccination, confounding by indication is driven by the perceived risk of COVID-19 outcomes and general health status, while frailty bias is more about the inherent vulnerability of individuals being vaccinated.

### 2.7 Differential depletion of susceptible bias

Differential depletion of susceptible (DDS) bias occurs when the most susceptible individuals are disproportionately removed from the at-risk population over time through infection or vaccination. Initially, highly susceptible individuals are more likely to contract COVID-19, making VE appear high. Over time, as susceptible individuals gain immunity, both vaccinated and unvaccinated groups show lower infection rates. This results in an apparent waning of VE, not due to the vaccine losing effectiveness but because the overall population has become less susceptible. In COVID-19 VE studies, this bias can underestimate VE over time, falsely suggesting waning VE. This bias is a form of selection bias because the composition of the population changes, affecting the observed effectiveness. DDS could affect all observational study designs used for VE studies. During the COVID-19 pandemic, many studies have shown waning effectiveness (41). For example, a study by Chemaitelly et al. from Egypt estimated the long-term effectiveness of COVID-19 mRNA boosters and reported negative relative effectiveness 6 months after boosting, attributing it to “negative immune imprinting” (42). However, Noam argued that in this study, it is possible that the use of discrete-time hazards conditioned on survival for at least 6 months after vaccination results in selection bias due to the depletion of susceptible from the cohort that did not receive a booster (43). Using a simulation model, Kahn et al. demonstrated that if baseline VE is high, the effect of depletion of susceptible bias is low. However, for 'leaky vaccines' (low baseline VE), the impact of this bias is higher (44). It is essential to consider the differential depletion of susceptible in vaccine effectiveness studies to avoid misleading conclusions.

## 3 Attrition bias

Attrition bias occurs when there are systematic differences between individuals who remain in a study and those who drop out over time (45). For instance, individuals from lower socioeconomic backgrounds, who often have different health behaviors, poorer health outcomes, and limited access to healthcare, are more likely to leave the study or not visit health facilities for diagnosis. This selective dropout can result in a non-representative sample, as these individuals are frequently at a higher risk of infection due to their circumstances. If they drop out disproportionately, the remaining participants may exhibit a lower overall risk of infection, potentially leading to an overestimation of effect size.

Attrition bias in observational VE studies is unique because these studies often involve large cohorts using secondary data. In many countries, COVID-19 vaccination was offered universally, and vaccination records are uniquely maintained in immunization registries, making the data on vaccination status better than other health data (46). However, outcome data for the cohort studies come from different registries, including hospitals, insurance, or similar databases, which can be linked to vaccination registries (47–49, 103). Access, affordability, and coverage of diagnosis and treatment may differ from vaccination, leading to a 'loss to follow-up' from the original cohort. Unlike prospective cohort studies, where investigators are aware of loss to follow-up, data linkage studies have no definitive way to determine whether there was loss to follow-up. Consequently, lost follow-up is often not reported in cohort studies on VE. For example, in the systematic review of Law et al. none of the cohort studies investigating the VE of inactivated vaccines reported the attrition rate (50). Discussing the impact of attrition on VE is specifically important based on the context and policies, health-seeking behaviors, and vulnerable groups.

## 4 Information biases

In VE studies using cohort design, information biases can significantly distort the VE estimates. These biases arise from misclassification of exposure or outcome, often due to inconsistent or inaccurate data collection methods. Once the cohorts are identified, it is crucial to obtain accurate data; however, measurement errors can lead to bias in the analysis, known as information bias (51). For example, variations in diagnostic testing practices, inaccuracies in vaccination records, or differential recall between vaccinated and unvaccinated individuals can result in either overestimation or underestimation of VE. The direction and magnitude of this bias depend on whether the distribution of errors for a specific variable, such as vaccination status or disease incidence, is influenced by the true values of these variables or by errors in measuring other variables.

### 4.1 Immortal time bias

Immortal time bias occurs in observational studies when an “immortal” time period is incorrectly classified or excluded from the analysis (52). This period is immortal because, during this time, the outcome of interest (such as infection or death) cannot occur. In COVID-19 vaccine studies, this bias can arise if the time between the initiation of the study and when individuals receive the vaccine is not correctly accounted for. If this period is mistakenly included in the vaccinated group, it can falsely enhance the vaccine's apparent effectiveness because individuals cannot experience the outcome during this “immortal” period. As an example, Flacco et al. (53) reported that the monthly mean death rates were 0.97 per 1,000 for vaccinated individuals and 2.26 per 1,000 for unvaccinated individuals, showing a significant difference in overall deaths. Their data indicates that the mean follow-up time was 561 days for those never vaccinated, compared to 399 days for those vaccinated, due to the time gap between the start of the study and vaccination. The vaccinated group had a mean of 162 days of immortal time before vaccination (only those still alive received the vaccine), while all deaths during this period were allocated to the unvaccinated group. Since this was a single cohort from a province, the vaccinated group contributed to the denominator of the unvaccinated group before receiving their vaccines. Using the same data, Berrino et al. (54) recalculated the mortality rates and concluded that the rates were almost similar when accounting for the immortal time of the vaccinated group. Immortal time bias is not due to differential vaccination but rather an error in the denominator, which may happen in the analysis process or retrospective cohort studies.

### 4.2 Misclassification bias

Misclassification bias occurs when individuals or events are incorrectly categorized regarding exposure or outcome status. This bias can arise if vaccination status or disease outcome is inaccurately recorded. While the misclassification of exposure status (vaccination) might seem theoretical in prospective cohort studies, most VE studies use retrospective designs. Due to incomplete or inaccurate historical data and errors in data linkage, there is a risk of misclassifying vaccination status. This is particularly relevant if vaccination records are not up-to-date or come from different healthcare providers with varying record-keeping practices. One of the first systematic reviews on the VE of COVID-19 vaccines showed that of the 42 studies published within the first 6 months, 31 used vaccination registries, five included self-reporting, and 6 did not report the source of vaccination information, highlighting the risk of misclassification bias in these studies (17).

Misclassification of disease or outcomes is expected due to variability in diagnostic test accuracy, differences in test administration timing, and potential misinterpretation of results. The COVID-19 pandemic led to rapid advancement and large-scale production of various diagnostic test kits, none of which possess 100% sensitivity or specificity. This inevitably results in false positives and false negatives, contributing to misclassification. Several systematic reviews on COVID-19 diagnostic tests indicate significant variability in sensitivity and specificity across different platforms, tests, timing of testing relative to exposure, and result interpretation (55–57). A meta-analysis of 24 commercially available antigen kits demonstrated a pooled sensitivity of 77% and a pooled specificity of 98%, highlighting a substantial risk of misclassification (58). Despite WHO's recommendation to use RT-PCR for VE studies (8), the reliance on two-stage screening procedures increases the likelihood of misclassification. These variations can lead to incorrect disease status classification, further complicating VE estimates.

During the pandemic, changes in diagnostic criteria (59–61), referral procedures, breakthrough infections, and even COVID-19 death classifications occurred over time. Since VE studies rely on real-world data, these variations in disease diagnosis and death classifications can differ not only over time but also across different sites, hospitals, or provinces, influenced by policy changes or subjective human factors. Many studies across the globe show that a large number of COVID-19 deaths are unaccounted for and reported as “excess mortality,” and there are time and space variations of these numbers (62). Surveillance and reporting biases further complicate the scenario; inconsistent case reporting practices and variability in public health surveillance intensity can lead to uneven detection and reporting of cases. Understanding the changes and events in study areas, regions, and countries over the study period is essential to comprehending misclassification biases.

#### 4.2.1 Non-differential misclassification of outcomes

Non-differential misclassification occurs when the misclassification rate is similar across groups. This can happen if the outcome measured has a low specificity, leading to false positive cases in both groups. It generally biases the results toward the null, diluting the observed effect size and making it harder to detect a true association between vaccination and outcomes (63). Sometimes, it can happen due to the way the “outcome” is recorded. This has been shown in studies where the allocation of outcomes (COVID-19-related hospital admission) uses data from the billing process. These include patients without symptoms admitted for other reasons but tested positive for COVID-19 (38). Because the outcome documentation is from billing data, those without clinical disease but positive test results were included as those with outcomes (COVID-19 hospital admissions). The misclassification is similar for both groups; thus, the VE estimates are diluted. Similarly, a study from the Netherlands showed that 42% of cases included in the hospital register on COVID-19 patients are missing the reason for admission, and whether the positive COVID-19 test results were associated with COVID-19 clinical disease is not known (103).

#### 4.2.2 Differential misclassification of outcome

Differential misclassification occurs when the likelihood of misclassification differs between groups (vaccinated vs. unvaccinated). Depending on the direction of the misclassification, it can lead to overestimation or underestimation of VE. In studies using secondary data for VE studies, the allocated diagnosis could be systematically different in the two groups. Some studies show that vaccinated people usually attribute mild to moderate symptoms to vaccine side effects and do not seek care, thus less likely to be diagnosed as having COVID-19 (38). On the other hand, healthcare workers may suspect COVID-19 more among unvaccinated groups during hospital visits and perform testing, leading to higher detection or “diagnostic bias.” This could be partly due to policies where test results were mandated for many institutions and procedures if the individual is not vaccinated, thus leading to more cases of asymptomatic or mild cases of COVID-19 among unvaccinated groups. This will lead to differential misclassification of outcome status among vaccinated and unvaccinated groups, inflating VE estimates. However, it will not always overestimate VE. A study in Australia reported that fully vaccinated participants were twice as likely as those who were unvaccinated to report positive COVID testing intentions, which may lead to overdiagnosis of COVID-19 in the vaccinated arm, underestimating VE (64). The context-specific factors could play a major role in deciding in which direction the bias operates.

### 4.3 Case counting window bias

The case counting window bias occurs when the time frame for counting cases in a study is not properly aligned with the period during which a vaccine is expected to be effective. This can happen if the cases are counted from the start of follow-up for the unvaccinated group but only after vaccination for the vaccinated group; the difference in these windows can lead to biased VE estimates. So, the cases in the unvaccinated group are counted during a period when they were at higher risk. In contrast, cases in the vaccinated group are only counted after they might have already benefited from some protection. Fung et al. showed that even a vaccine with almost zero VE could be presented as having a VE of 48% if the case counting window bias is ignored (65).

### 4.4 Waning immunity bias

Waning immunity bias occurs when the observed effectiveness of a vaccine appears to decline over time due to a natural decrease in immune response, notably the reduction in neutralizing antibody levels. Initially, vaccines demonstrate high effectiveness due to a robust immune response. However, as antibody levels wane, individuals may become more susceptible to infection, creating the perception of declining vaccine effectiveness (VE). Numerous studies, including several systematic reviews (45, 66, 67), have reported this phenomenon, particularly those involving mRNA vaccines against COVID-19. A systematic review of 40 studies estimated that VE against omicron infection and symptomatic disease decreased by 20% at 6 months post-primary vaccination cycle and by 30% at 9 months post-booster dose administration (68). This waning immunity is a natural biological process; failing to account for it in VE studies properly can introduce bias, leading to inaccurate interpretations. Long-term VE studies must incorporate original or short-term VE data and clearly state the duration post-vaccination to avoid biased interpretation of VE estimates. Additionally, considering the date of vaccination in analysis and data presentation is essential. Addressing this bias is crucial, particularly in the ongoing “infodemic” (69) and vaccine hesitancy.

## 5 Confounding

Confounding occurs when an extraneous factor influences both the likelihood of vaccination and the health outcome, leading to a distorted estimate of vaccine effectiveness (18). This manuscript has described factors such as health-seeking behaviors, demographic differences, and underlying health conditions leading to differential vaccination. While these factors were labeled as biases—such as selection bias, healthy user bias, and frailty bias—these are, in fact, confounding factors. They influence both vaccination status and outcomes, distorting the true effect of the vaccine. For instance, individuals with prior exposure to SARS-CoV-2 or underlying comorbidities may have different risks of severe COVID-19, which could distort VE estimates if not properly adjusted for (70, 71, 100). In addition to these specific factors, researchers must consider other confounders that may further affect VE estimates, such as geographic differences, occupational exposures, and previous infection history. Failure to control for such confounders can lead to inflated or underestimated VE estimates, particularly when assessing protection against severe disease. To ensure accurate and reliable VE estimates, thorough identification and adjustment of these confounding factors are essential in observational studies.

## 6 Biases and challenges of VE studies in LMICs

The assessment of VE in low and lower-middle-income countries (LMICs) faces significant challenges due to various biases and unique factors. Disparities in vaccine deployment, reliance on less effective vaccines, supply constraints, and dosing challenges necessitate evaluations of mixed and suboptimal regimens. Additionally, distinct health and demographic profiles can lead to differential vaccination coverage, altering estimated vaccine protection (72). High seroprevalence of naturally acquired immunity and the risk of new variant emergence due to uncontrolled transmission further complicate VE estimates. The absence of robust systems for maintaining vaccination records, integrating data across platforms, and ensuring accurate digitalization, along with limited diagnostic capacity and barriers to accessing and affording healthcare, exacerbate the risk of misclassification of both exposure (vaccination status) and outcome (disease occurrence). Such misclassification is a critical source of information bias in these studies, significantly impacting the accuracy of VE estimates. Therefore, targeted VE studies are essential to develop accurate and effective vaccination strategies tailored to LMICs. However, the availability of vaccine studies in these settings is limited. A systematic review by Petráš et al. included 761 published VE studies, but only two were from low-income countries, both from Zambia (73). One was from a prison outbreak using a case-control design with self-reporting/rapid tests (74), and the other was a hospital-based study with significant missing data (75). Among lower-middle-income countries, Indian authors published 28 studies. Except for India, only Bangladesh (76), Egypt (77), Morocco (78), and Pakistan (79–83) had estimated VE in their settings. Real-world evidence and the effect of biases on those estimates from LMICs are missing in global literature. A comprehensive analysis of these available studies is critical to understanding how specific biases affect VE estimates in future academic preparations.

## 7 Effects of biased estimates

Biases significantly impact the internal validity of vaccine efficacy (VE) studies, with far-reaching societal and policy implications. Early studies often reported VE as high as 95% (84), fostering overconfidence in vaccine protection and prompting global policy changes. Health agencies described vaccines as “extremely protective,” creating a misconception that vaccines could prevent infection (85). This led to policy changes in some places prioritizing vaccination for transmission interruption, sometimes neglecting vulnerable populations at higher risk for severe COVID-19 outcomes. Overestimating VE altered public perception, with increased infection rates post-vaccination due to risk behaviors (86). Variations in VE estimates influence vaccination willingness (87); one study showed that 51.3% would accept a COVID-19 vaccine that is 50% effective, and 77.1% would accept a vaccine that is 95% effective (84). Differences in VE estimates can significantly fuel vaccine hesitancy and erode public trust in vaccination programs. Media coverage and anti-vaccine movements can exploit these inconsistencies, spreading misinformation and increasing hesitancy. Transparent communication about the limitations and strengths of VE studies is crucial to maintaining public trust and encouraging vaccine acceptance.

## 8 Minimizing biases in cohort studies on VE

Target trial emulation (TTE) is often used to minimize biases in observational studies on VE by applying design principles from randomized trials to the analysis of observational data, thereby explicitly tying the analysis to the trial it is emulating. The TTE approach was extensively used in COVID-19 VE studies when applying cohort design (14, 88–94, 102). Still, the challenges and errors in properly executing this approach can introduce the abovementioned biases, resulting in inaccuracies in estimating VE if the emulation is improperly planned and executed. Although referred to as **“**TTE bias” in literature, it is not technically a specific type of bias but could be due to any other type detailed above.

Biases, once introduced, are challenging to control in observational studies. Minimizing bias during the design stage is paramount. The WHO guidelines on observational studies for COVID-19 vaccine effectiveness (8) outlined various strategies to mitigate these biases. Bayesian modeling approaches could address specific biases, such as misclassification bias due to imperfect tests (95). A thorough evaluation of all potential biases is essential when reporting VE studies but reporting and assessing the direction of biases may be more challenging than it seems. Brookmeyer and Morrison (96) demonstrated this complexity using data published in a VE study (97) to simulate different biases occur in linked registry studies. They showed that if the bias is due to a single source, the direction of the bias is predictable. However, if multiple sources of biases are present, then the direction of the bias can be either way. Often, the biases are multiple; thus, predicting direction can be difficult. It is crucial to discuss these biases' probable impact and direction on VE study results and report them comprehensively in all observational studies.

## 9 Conclusion

These well-known epidemiological biases may occur more frequently during a pandemic. Rapid vaccine development and distribution can lead to differential vaccination practices, prioritizing high-risk populations and exacerbating frailty bias and confounding by indication. Socioeconomic disparities and diverse health behaviors become more pronounced, while the overburdened health systems increase the risk of data inaccuracies and outcome misclassification. The frequency of occurrence of these biases varies widely based on the setting, data sources, and analysis. These biases could be higher in VE in LMIC settings due to a lack of proper data sources. Understanding and mitigating these biases are crucial for accurate VE estimates, informing public health strategies, and maintaining public trust in vaccination programs. With the peak pandemic now behind us and less urgency for rapid publication, a comprehensive investigation into the long-term vaccine effectiveness is warranted, ensuring that all previously discussed biases are thoroughly addressed. Additionally, future studies must explicitly account for whether individuals who were infected with COVID-19 subsequently received vaccination, as this may affect long-term outcomes such as mortality and PASC. While studies like Cai et al. (98) demonstrate that the risk of death declines over time but remains elevated in previously hospitalized patients, they do not account for whether subsequent vaccination modifies this risk. Incorporating this factor in future research will provide more accurate assessments of vaccine effectiveness and long-term health risks, thereby guiding public health policies more effectively.
